# [Corrigendum] DOCK8 interference alleviates Aβ‑induced damage of BV2 cells by inhibiting STAT3/NLRP3/NF‑κB signaling

**DOI:** 10.3892/etm.2025.12817

**Published:** 2025-02-06

**Authors:** Xueying Zhou, Ji Hu, Deyi Xu, Sheng Zhang, Qianyan Wang

Exp Ther Med 25:134, 2023; DOI: 10.3892/etm.2023.11833

Subsequently to the publication of the above article, the authors have contacted to Editorial Office to explain that the data shown for scratch-wound assay experiments in Fig. 7A and the Transwell assay data in Fig. 7B on p. 5 were inadvertently chosen incorrectly for this figure. The authors, however, had retained their original data, and the revised version of Fig. 7, now showing the intended data for these experiments (and re-plotted bar charts related to these data), is shown below. Note that the errors made in compiling Fig. 7 did not have a major impact on either the overall results or on the conclusions reported in this study. All the authors agree with the publication of this corrigendum, and are grateful to the Editor of *Experimental and Therapeutic Medicine* for granting them the opportunity to publish this; furthermore, they apologize to the readership for any inconvenience caused.

## Figures and Tables

**Figure 7:**
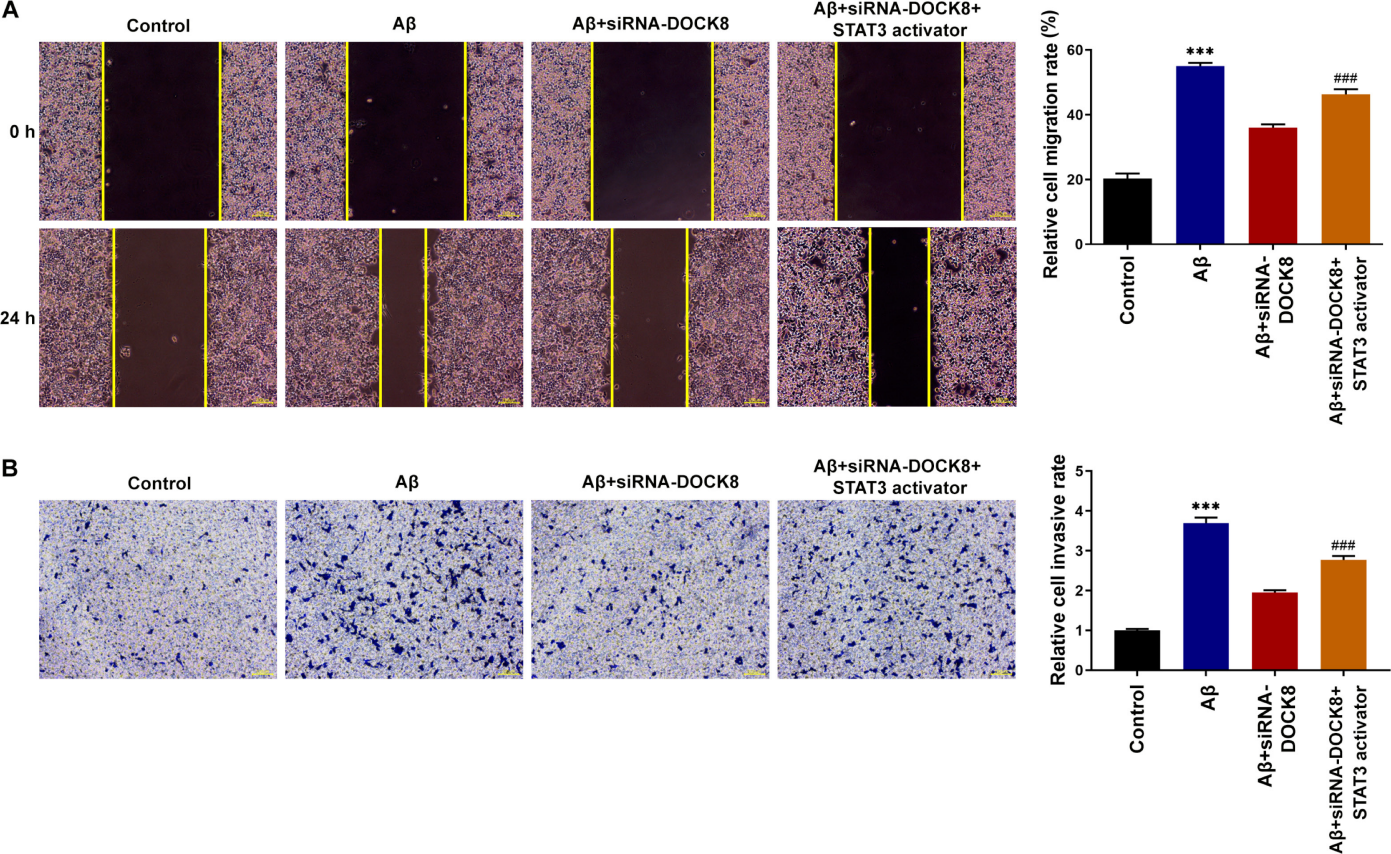
DOCK8 interference inhibits the migration and invasion of Aβ-induced BV2 cells by suppressing STAT3/NLRP3/NF-κB signaling. (A) The cell migration was detected using wound healing assay. Magnification, x100. (B) The cell invasion was detected using Transwell assay. Magnification, x100. ^***^P<0.001 vs. control; ^###^P<0.001 vs. Aβ + siRNA-DOCK8. DOCK8, dedicator of cytokinesis 8; Aβ, amyloid β; NLRP3, NLR family pyrin domain containing 3; siRNA, short interfering RNA; NC, negative control.

